# A novel hybrid transformer-CNN architecture for environmental microorganism classification

**DOI:** 10.1371/journal.pone.0277557

**Published:** 2022-11-11

**Authors:** Ran Shao, Xiao-Jun Bi, Zheng Chen

**Affiliations:** 1 College of Information and Communication Engineering, Harbin Engineering University, Harbin, China; 2 College of Information and Communication Engineering, Harbin Vocational & Technical College, Harbin, China; 3 Department of Information Engineering, Minzu University of China, Beijing, China; Sejong University, REPUBLIC OF KOREA

## Abstract

The success of vision transformers (ViTs) has given rise to their application in classification tasks of small environmental microorganism (EM) datasets. However, due to the lack of multi-scale feature maps and local feature extraction capabilities, the pure transformer architecture cannot achieve good results on small EM datasets. In this work, a novel hybrid model is proposed by combining the transformer with a convolution neural network (CNN). Compared to traditional ViTs and CNNs, the proposed model achieves state-of-the-art performance when trained on small EM datasets. This is accomplished in two ways. 1) Instead of the original fixed-size feature maps of the transformer-based designs, a hierarchical structure is adopted to obtain multi-scale feature maps. 2) Two new blocks are introduced to the transformer’s two core sections, namely the convolutional parameter sharing multi-head attention block and the local feed-forward network block. The ways allow the model to extract more local features compared to traditional transformers. In particular, for classification on the sixth version of the EM dataset (EMDS-6), the proposed model outperforms the baseline Xception by 6.7 percentage points, while being 60 times smaller in parameter size. In addition, the proposed model also generalizes well on the WHOI dataset (accuracy of 99%) and constitutes a fresh approach to the use of transformers for visual classification tasks based on small EM datasets.

## 1. Introduction

Environmental Microorganisms (EMs) (e.g., bacteria, viruses, etc.) have a typical size range between 0.1 and 100 microns and thus cannot be detected with the naked eye. They can be classified as cell-free, multicellular, or unicellular species [1]. Despite their tiny size, EMs can have a significant impact on human life. On the beneficial side, some EMs are used to make fermented foods such as cheese and bread, break down plastics, treat sulfurous emissions from industrial processes, condition soil, digest organic waste from sludge, and enhance the quality of freshwater [2–4]. On the harmful side, some EMs can reduce crop yields, cause food spoilage, and will even lead to diseases such as the novel coronavirus disease 2019 (COVID-19) and death [5, 6]. Therefore, the correct identification of EMs is necessary for the study of their beneficial or adverse effects on other organisms and the environment [7, 8].

Due to their small size, the classification of EMs is difficult [9]. The two traditional classification methods typically used for EM classification are molecular biology (e.g., DNA and RNA analysis), and morphological approaches (e.g., manual observation under a microscope) [10]. The former has high accuracy but requires expensive equipment, while the latter requires a significant amount of time and effort on manual labor by a professional researcher [11, 12]. As a result, many researchers have attempted to classify images using computer-aided methods based on machine learning [13, 14], which has allowed the development of classification methods that are efficient and do not require substantial expertise. However, these methods still require substantial feature engineering to formulate classification conditions and cannot extract the EMs’ salient features automatically for classification [15].

Due to the rapid development of artificial intelligence in recent years, deep learning-based methods are being adopted for automated salient feature extraction and end-to-end classification. Such methods have thus emerged as primary tools for image classification [16–18]. For EM image classification tasks, several researchers have employed deep learning methods based on convolutional neural networks (CNNs) [19, 20]. However, although CNNs have been shown to be effective in extracting local image features, their performance is lacking when it comes to capturing long-distance dependencies [21]. It should be noted that for 2D image classification tasks, locality and long-distance dependencies have been shown to be equally important [22, 23]. Due to their capacity to capture long-range dependencies, transformers [24, 25] are now commonly used in modern deep learning applications, including computer vision (CV) [26, 27] and speech processing [28]. Transformers were first proposed as components of machine translation models [29]. The vision transformer (ViT), the first pure transformer architecture used in CV, was proposed only recently [30]. In terms of image classification, it achieves results that are competitive with many state-of-the-art CNNs [31, 32]. Zhao P. et al. [33] first applied ViTs, such as ViT [30], DeiT [34], T2T-ViT [35] and BotNet [36], to EM classification tasks. However, when trained on the sixth version of the EM dataset (EMDS-6) [37], which is a small dataset consisting of only 21 classes and 1680 EM images, these models’ performance was inferior to that of similar-sized CNNs, such as Xception [38]. The reasons for this are twofold. First, due to the constant patch size of ViTs, it is extremely difficult for the transformer to extract multi-scale feature maps definitively, which presents a significant challenge for classification tasks [39]. Second, ViTs lack the locality inherent in the design of CNNs, which makes them unsuitable for image classification problems on small datasets like EMDS-6 [30].

To enhance ViT performance and raise it to a level comparable to that of the state of the art using small EM datasets, in this work, a novel hybrid transformer-CNN EM classification architecture (HTEM) is proposed. To address the first of the two main issues discussed above, a hierarchical structure is adopted to obtain multi-scale feature maps. For the second problem, additional convolution operations are introduced in the transformer’s two core sections, which makes the HTEM inherently efficient, both in terms of number of parameters and computational efficiency.

In the proposed HTEM model, the transformer is divided into four stages to create a hierarchical framework, and all stages share a similar structure. The input images first go through a convolution token embedding (CTE) block for feature extraction [40, 41]. The resulting data of the CTE block are fed into a feature-embedding layer to reduce the size of feature maps. This embedding layer allows the generation of different scales of images for the classification task, while also reducing the number of parameters and thus the computation load incurred by the network. The 2× down-sampled feature maps are then input to the *i*-th stage transformer encoder, which consists of a convolutional parameter sharing multi-head attention (CPSA) block and a local feed-forward network (LFFN) block. Inspired by [42], the linear projection before each self-attention block in the original transformer model is replaced by the convolutional parameter sharing projection in the CPSA block. This allows the key and value matrices of the original transformer model to share the same parameters and perform depth-wise separable convolution operations [43] to subsample the feature maps. By subsampling and sharing matrix parameters, the block can capture more local features for image classification and simultaneously minimize the computational complexity and excessive flexibility of the transformer model, thus increasing efficiency with minimal performance loss. Additionally, motivated by [44], the LFFN block is proposed as a replacement for the feed-forward network (FFN) in the transformer model. This block introduces a sandglass block with additional depth-wise convolutions in the FFN block to help the transformers capture more local features. These depth-wise convolutions provide an effective mechanism for local content aggregation, which is not available in the traditional ViT’s FFN block. It should be noted that the depth-wise convolution is effective in terms of both parameters and computation complexity. The basic transformer encoder structure is repeated multiple times at each stage. After four feature embedding layers and encoding stages, a global average pooling block is used to replace the class token in the transformer model for better classification results.

Overall, the proposed HTEM bears all the advantages of CNNs and transformers through its efficient hierarchical network structure and the introduction of improved local feature perception. The results show that the proposed model achieves state-of-the-art performance while remaining lightweight and efficient when trained on a small EM dataset like EMDS-6. Additionally, the proposed HTEM outperforms all compared CNN- and transformer-based models; after data augmentation, its accuracy is 9.02 percentage points higher than Xception while requiring less training time and fewer parameters. Additionally, it is demonstrated that the proposed model’s performance is not contingent on the presence of the token position embedding. This allows for a simplified architecture design and offers flexibility with respect to the input images’ resolutions, which is essential for classification tasks.

To summarize, the main contributions of this paper are three-fold:

1. We put forward a new hierarchical transformer architecture called HTEM, which can capture multi-scale feature maps of EM images, thus strengthening the model’s ability to capture salient features.
2. We introduce the CPSA and LFFN blocks to the transformer’s core sections to increase the number of convolutions. The novel model architecture can simultaneously capture local features and long-range dependency information and is free of any position embedding dependence.
3. Experimental results on the EMDS-6 and WHOI datasets show the effectiveness and generalization ability of HTEM with less training time and a lower number of parameters compared with previous state-of-the-art ViTs and CNNs.

The rest of this paper is organized as follows. A brief overview of EM image classification methods and ViTs is presented in Section 2, while the details of the proposed HTEM are presented in Section 3. In Section 4, the training details are described and comprehensive experimental comparisons are discussed. Finally, the paper is concluded in Section 5.

## 2. Related works

### 2.1 EM image classification

With the development of computer and imaging technology, computer-aided EM classification based on machine learning has achieved remarkable results. Kruk M. et al. [45] proposed a system for classifying soil EMs using shape, edge, and color features, which uses a random forest to classify EM images. Cunshe C. et al. [46] proposed a method for classifying wastewater EMs using morphological features combined with principal component analysis. Xiaojuan L. et al. [47] proposed a method for rapid classification and identification of bacteria in wastewater using edge detection. Li C. et al. [48] proposed an EM classifier that captured edge features and was based on a support vector machine. Although the above methods are able to perform EM classification and identification, they require extensive feature engineering for assisted learning and are not able to achieve automatic end-to-end classification.

In order to mitigate the reliance on feature engineering, more and more researchers have adopted deep learning methods based on CNNs for EM classification tasks. Luo et al. [49] used CNNs for classification studies of planktonic microorganisms. Połap et al. [50] proposed a CNN model based on regional covariance to classify EMs and they demonstrated the classification of rod-shaped and spherical bacteria with accuracies higher than 91% and 78%, respectively. Bliznuks D. et al. [51] proposed a bacterial growth analysis system using a 3D CNN, which also achieved high accuracy. Zihan Li Z. et al. [52] presented the fifth version of the environmental microorganism dataset (EMDS-5) and used the VGG and InceptionV3 networks to classify microorganisms. Recently, Zhao P. et al. [37] proposed the EMDS-6 dataset and conducted an EM classification study using typical CNNs.

However, it is known that CNNs are ineffective in capturing long-distance dependencies in EM images [30]. Due to the better ability of transformers in capturing long-distance dependencies, Zhao P. et al. [33] first proposed the use of ViTs for an EM image classification study on the EMDS-6 dataset, which was a novel approach compared to others presented by that time. The difference with the present work is that in this study a hybrid transformer-CNN architecture is proposed instead of ViTs. This model combines the respective advantages of CNNs and transformers to realize the EM image classification task on the EMDS-6 dataset.

### 2.2 Visual transformers

The first instance of transformers being used for CV tasks was ViT. This model achieves state-of-the-art results when the corresponding dataset is sufficiently large, such as in the case of ImageNet-22k, JFT-300M, etc. Subsequently, many models have been proposed in order to obtain better results in image classification tasks [34, 53, 54]. For instance, Tokens-to-token (T2T-ViT) [35] uses additional sliding windows to integrate multiple tokens, an operation that increases the ability of the model to obtain local information but increases its complexity. Recently, some efficient models have been proposed to avoid the reliance of ViT models on large datasets. For instance, CeiT [41] first uses convolution to capture the input image features, and then captures more local information through the integration of the depth-wise convolution module into the ViT. ConViT [55] utilizes gated position self-attention to simulate the effect of convolutional operations so that the model can obtain the local contents. CvT [42] enhances the ViT’s local feature processing capability by means of convolution operations introduced in both linear projection and multilayer perceptron blocks.

These improvements allow the ViT to perform well even in the absence of large datasets, and the models mentioned have achieved better results on medium-sized datasets (e.g., ImageNet) [56]. However, for small datasets, the above models still fall behind similar-sized CNNs. The key contrast between the proposed work and previous research is that a hybrid transformer network is put forward, comprising a hierarchical structure and incorporating the convolutions in the transformer’s core sections so that it can be trained effectively and efficiently even on small datasets like EMDS-6.

## 3. Methods

### 3.1 Overall architecture

**Fig 1:**
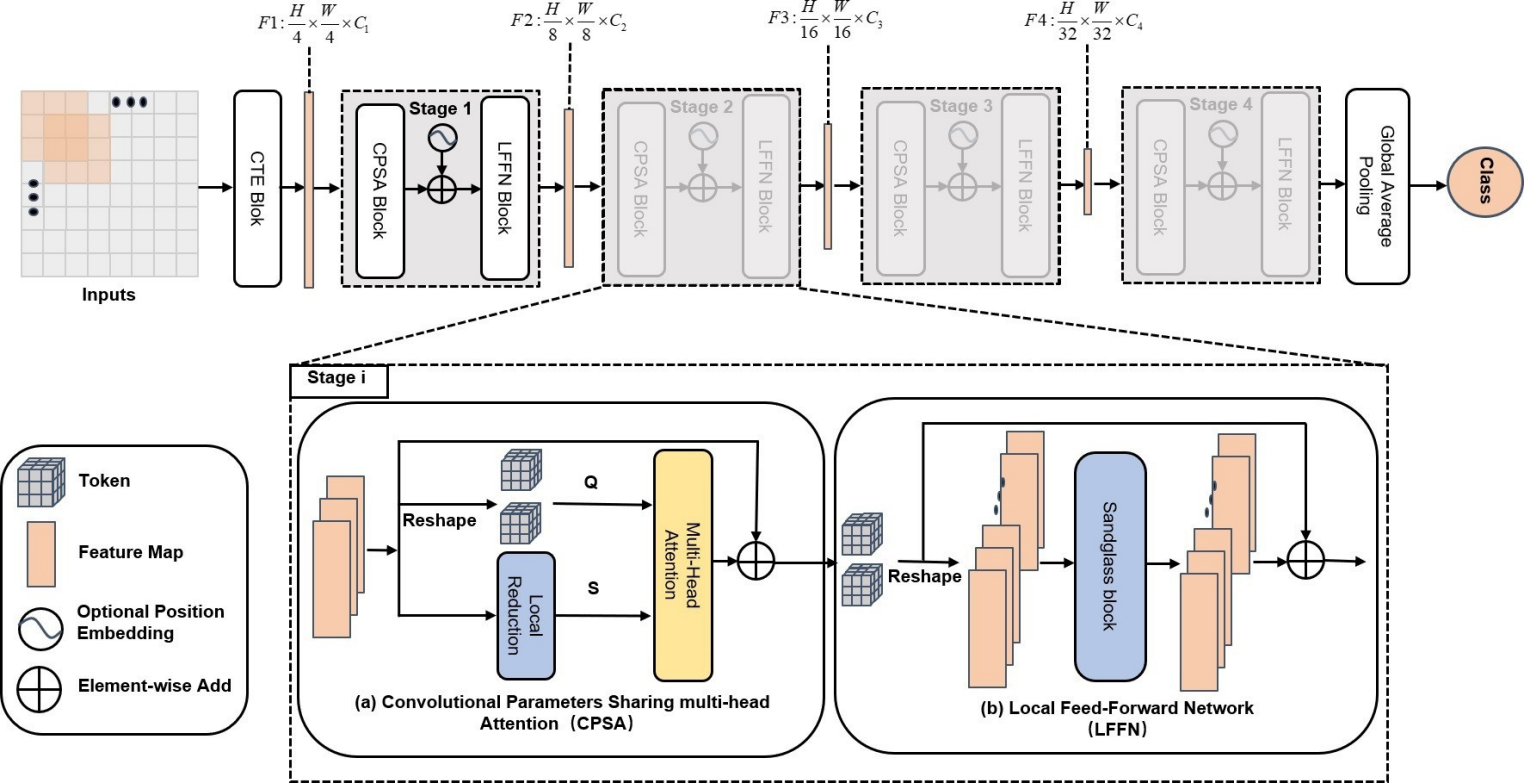
Overall architecture of HTEM. First, the inputs are fed to a Convolutional Token Embedding (CTE) to obtain patches. Second, the feature maps are processed using a feature embedding layer and repeated transformer-encoder blocks consisting of CPSA and LFFN blocks in each stage. Finally, a global average pooling block is employed to obtain the class token.

The aim of this work is to present a new hierarchical transformer architecture with more convolution operations in the transformer’s core sections so that it can effectively classify data after training using small datasets. Fig 1 shows an overview of the proposed HTEM model. In this work, a multi-stage structure is used, where all stages utilize a similar structure comprising of a CPSA block and an LFFN block.

One of the major differences of the proposed architecture is that it does not employ a fixed patch size for token embedding as ViT does. Instead, a CTE block is utilized, consisting of a convolution operation with a stride of 1 and an output channel of 16, followed by ReLU activation and a maxpooling layer with a stride of 2 to extract local information efficiently. Inspired by the layout of CNNs, in order to extract the multi-scale feature maps, before each level a feature embedding layer comprising a convolution operation is used to reduce the size of the intermediate feature maps (2×downsampling of resolution) and project it to a higher dimension. From an input image, the proposed model produces four hierarchical feature maps with strides of 4, 8, 16, and 32. With the above feature maps’ hierarchical architecture, the model can extract multi-scale representations, which makes it more suitable and computationally efficient for EM image classification. In each stage, several similar encoder blocks, comprising a CPSA block and an LFFN block, are consecutively layered for feature transformation while maintaining the resolution of the input feature maps. The encoder block not only reduces the computational complexity and the over-flexibility of the transformer model but also simultaneously captures both the local contents and long-range dependencies. A global average pooling layer, a fully connected layer, and a softmax classification layer make up the model’s final block. In addition, this model does not require any position embedding to aid the training process.

In the following, we first demonstrate the implementation of a novel projection method using the CPSA block. Then, we describe the LFFN block and how its efficient design boosts the performance of the network.

### 3.2 Convolutional Parameters Sharing Multi-head Attention (CPSA)

**Fig 2:**
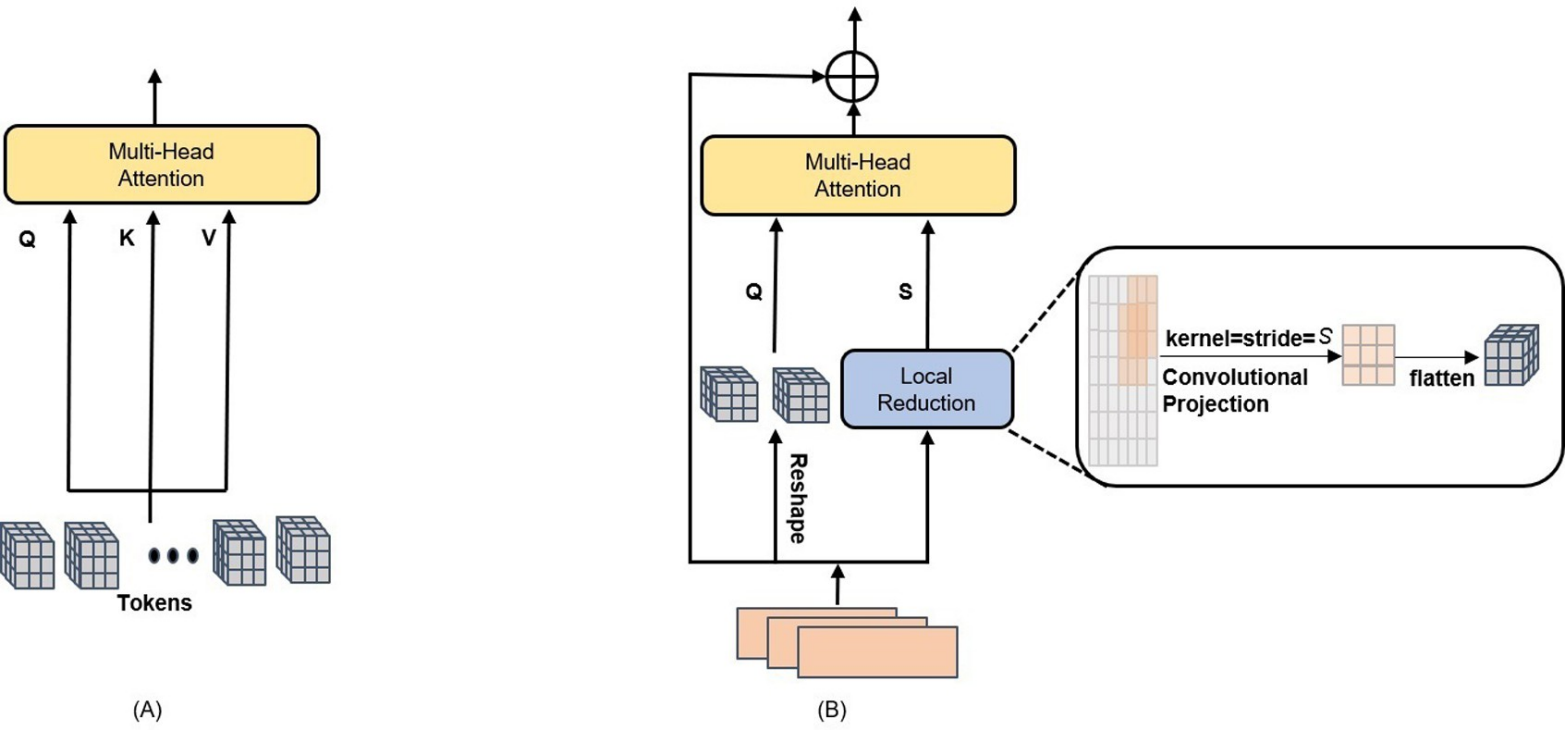
(A) Multi-Head Attention (MHA) block in ViT [30]. (B) Convolutional Parameters Sharing multi-head Attention (CPSA) block in HTEM.

In order to allow the proposed HTEM to capture more local features while at the same time maintaining computational efficiency, in this paper the adoption of a CPSA block is proposed instead of the multi-head attention (MHA) block employed in the original transformer architecture. Fig 2A shows the original MHA block, which employs a linear projection to extract the attention feature, while Fig 2B shows the proposed CPSA block, which uses the convolutional parameters’ shared projection to calculate the attention score of the model.

In order to decrease the complexity of the HTEM model and achieve high accuracy, the sharing between the key and value is enabled, which results in the shared parameter matrix *S*. This means that the proposed CPSA block employs a query *Q*, a shared parameters matrix *S* to replace *Q*, a key *K*, and a value *V* to obtain an attention feature. First, the CPSA block uses a convolutional projection to reduce the dimensions of input feature maps. The convolutional projection is implemented through a depth-wise separable convolution with a kernel size of *s* and a stride of *s*. This operation enhances the ability of the model to capture local information while improving its computational efficiency. Then, the tokens are compressed into 1D sequence features, thus generating the shared parameters matrix for subsequent processing. This is expressed mathematically as follows:

$$S=Flatten(DWConv2d(x_{i},s,s))$$ (1)

where *S* denotes the shared parameter matrix used for calculating the attention feature, *x*_*i*_ denotes the input feature maps before the convolution projection, *DWConv2d* denotes depth-wise separable convolution operation, *Flatten* indicates the conversion of a 2D feature map to 1D sequence features, and *s* represents the size of the depth-wise convolution kernel and its stride, which in the following will be referred to as “reduction rates”.

Inspired by [30], in order to guarantee the consistent dimensionality of the output tokens, *Q* always maintains its original dimensions and is not processed through the convolutional projection. To obtain *Q*, the feature maps are reshaped from 2D to 1D feature maps. Then, the relationship between the tokens is modeled through the similarity between the Q-S pairs, which results in the generation of the attention score, which can be mathematically expressed as follows:

$$Attention(Q,S)=Soft\text{max}\left(\frac{QS^{T}}{\sqrt{d_{head}}}\right)S$$ (2)

where Attention() denotes the output attention features after the attention operation, the *Soft*max function is applied to the rows of the similarity matrix and *d*_*head*_ denotes the dimension of each attention head.

The effectiveness of the proposed HTEM is ensured by three crucial factors in the CPSA block. First, the utilization of depth-wise separable convolution introduces only *s*^*2*^*C* additional parameters compared to MHA in the original transformer, which is negligible compared to the total number of parameters in the model. Second, matrix sharing reduces the number of key and value parameters in half. This approach results in a diminished network learning capacity, but in the case of small EM datasets like EMDS-6, it also prevents overfitting. Third, each convolution projection operation is analogous to a matrix downsampling operation. Consequently, the number of *S* parameters is decreased by a factor of *s*, and the computational cost of the later corresponding multi-headed attention mechanism is lowered by a factor of *s*^*2*^.

### 3.3 Local Feed-Forward Network (LFFN)

**Fig 3:**
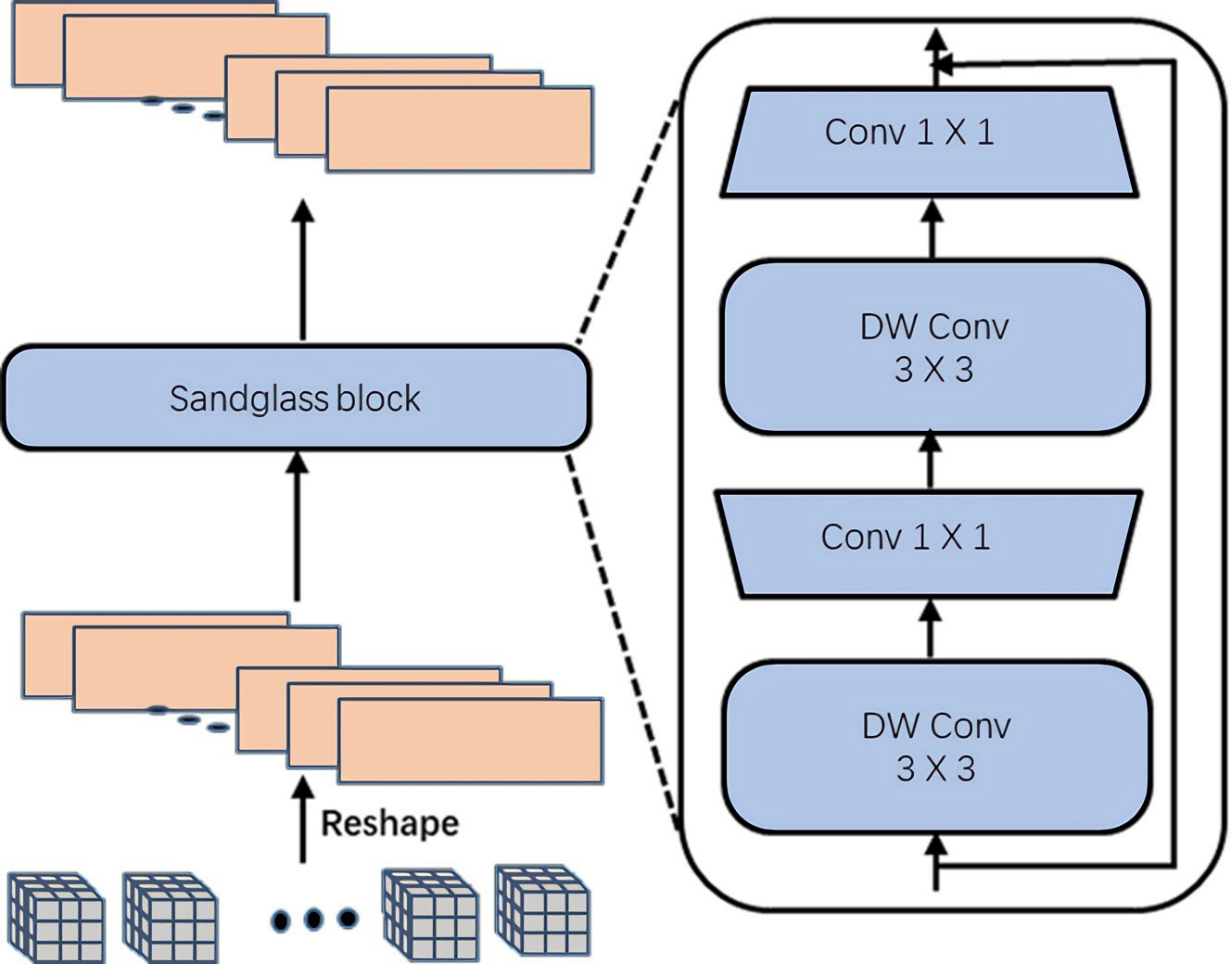
Local Feed-Forward Network (LFFN) in HTEM. DW Conv denotes depth-wise convolution.

In the original Transformer, an FFN block is appended after the attention layer. However, the FFN block only utilizes two fully-connected layers to achieve feature representation and cannot capture the correlation among neighboring tokens in the spatial dimension. Therefore, it would be useful if local dependencies could be effectively added to the fully connected network. The expansion of the hidden dimension between fully-connected layers is reminiscent of the sandglass block in MobileNeXt [44]. As shown in Fig 3, in this paper an LFFN block is proposed instead of the FFN block in the original transformer. The LFFN block allows the HTEM to capture more local features at different dimensions by introducing a sandglass block consisting of depth-wise separable convolution, thus improving the local information acquisition capability of the model.

The LFFN block performs the following operations. First, in order to improve the information interaction between adjacent pixels, the 1D feature inputs i.e., the tokens $x_{i}^{h}\in {\mathbb{R}}^{N\times C}$ produced by the CPSA block, are transformed to 2D features as shown in Fig 3. The feature representation is

$$x_{i}^{S}=\text{Re}shape2D(x_{i}^{h}),\;x_{i}^{s}\in {\mathbb{R}}^{\sqrt{N}\times \sqrt{N}\times C}$$ (3)

where $x_{i}^{s}$ denotes the transformed 2D features, *N* denotes the number of feature inputs, *C* denotes the feature dimension, and Re*shape2D* denotes the conversion of 1D sequence features to 2D feature maps. Second, in order not to lose low-dimensional local feature information, a depth-wise separable convolution with a kernel size of *k* is employed to process the 2D features. The computation could be represented as

$$x_{i}^{d(1)}=DWConv2d(x_{i}^{s}),\;x_{i}^{d(1)}\in {\mathbb{R}}^{\sqrt{N}\times \sqrt{N}\times C}$$ (4)

where $x_{i}^{d(1)}$ denotes the new feature maps after the depth-wise separable convolution operation *DWConv2d*. Then, the feature dimension is expanded using convolution with a kernel size of 1 to generate a new feature, i.e.,

$$x_{i}^{l(1)}=Conv(x_{i}^{d(1)}),\;x_{i}^{l(1)}\in {\mathbb{R}}^{\sqrt{N}\times \sqrt{N}\times (e\times C)}$$ (5)

where $x_{i}^{l(1)}$ denotes the feature maps after the dimension expansion, *Conv* denotes a convolution operation with a kernel size of 1, and *e* denotes the expansion ratio in the LFFN block. In order to obtain additional high-dimensional local contents, a depth-wise convolution is applied with a kernel size of *k*. That is,

$$x_{i}^{d(2)}=DWConv2d(x_{i}^{l(1)}),\;x_{i}^{d(2)}\in {\mathbb{R}}^{\sqrt{N}\times \sqrt{N}\times (e\times C)}$$ (6)

where $x_{i}^{d(2)}$ denotes the new higher-dimension feature maps after the depth-wise separable convolution operation. Finally, the output feature maps $x_{i}^{l(2)}$ of the LFFN block are generated through the application of a dimensionality reduction operation in the form of a convolution with a kernel size of 1. The operation can be represented as

$$x_{i}^{l(2)}=Conv(x_{i}^{d(2)}),\;x_{i}^{l(2)}\in {\mathbb{R}}^{\sqrt{N}\times \sqrt{N}\times C}$$ (7)

Note that the depth-wise convolutions introduce the most of the additional computational overhead of the LFFN block, but it is negligible compared to the total number of parameters in the whole model.

## 4. Results

In this section, the proposed HTEM model is evaluated using the EMDS-6 and WHOI datasets. Moreover, ablation investigations are conducted to confirm the efficacy of each component of the proposed architecture.

### 4.1 Experimental settings

#### 4.1.1 Network architecture

The proposed HTEM architecture used for the experiments of this study is presented in Table 1. The CTE block consisted of a convolutional layer with a kernel size of 3 and a stride of 1, and generated 16 enriched channels. To ensure stable training, a BatchNorm layer was also introduced. Then, a max-pooling layer was used with a kernel size of 3 and a stride of 2. The CPSA and LFFN blocks employed are shown in brackets, with the numbers of blocks stacked. In each CPSA block of stage *i*, *h*_*i*_, and *s*_*i*_ are the number of heads and reduction rates, respectively, while *e* denotes the expansion ratio in the LFFN block, and *k* is the depth-wise convolutions’ kernel size in the LFFN block.

**Table 1:** Detailed settings of HTEM.

Output	Layer Name	HTEM
*112*×*112*	CTE	Conv:3×3, 16, stride = 1 Maxpooling:3×3, stride = 2
*56*×*56*	Feature Embedding	2×2, 24, stride = 2
Stage 1	CPSA LFFN	$\left[\begin{array}{l} h_{1}=1,s_{1}=8\\ e=4,k=3 \end{array}\right]\times 2$
*28*×*28*	Feature Embedding	2×2, 32, stride = 2
Stage 2	CPSA LFFN	$\left[\begin{array}{l} h_{2}=2,s_{2}=4\\ e=4,k=3 \end{array}\right]\times 2$
*14*×*14*	Feature Embedding	2×2, 48, stride = 2
Stage 3	CPSA LFFN	$\left[\begin{array}{l} h_{3}=4,s_{1}=2\\ e=4,k=3 \end{array}\right]\times 4$
*7*×*7*	Feature Embedding	2×2,64, stride = 2
Stage 4	CPSA LFFN	$\left[\begin{array}{l} h_{4}=8,s_{4}=1\\ e=4,k=3 \end{array}\right]\times 2$

#### 4.1.2 Dataset description and preprocessing

In this experiment, the model performance is evaluated using the EMDS-6 and WHOI datasets.

EMDS-6 is an EMs dataset containing 840 distinct EM images in 21 categories of 40 images each. Because the EMDS-6 is a very small dataset, 37.5% of the dataset was selected as the training set, 25% as the validation set, and 37.5% as the test set. as in [33]. In the data augmentation experiment, the same five geometric enhancements as in [33] were adopted to enhance the EMDS-6 dataset, namely rotation by 90°, 180°, and 270°, as well as up-down and left-right mirror transformations. After data augmentation, the resulting dataset size was 6 times that of the original. In addition, following [33], in the imbalanced data experiments, after the data augmentation, each of the 21 types was viewed as a positive sample in turn, while the remaining 20 types of samples are considered negative samples. In this way, 21 new imbalanced datasets were obtained and used to validate the performance of the proposed model on imbalanced datasets.

WHOI is a dataset of cells and other planktonic particles that Imaging FlowCytobot collected from the water at Woods Hole Harbor. The dataset includes 6600 manually categorized photos that have been divided into equal-sized training and testing sets. There are 22 categories of photos, and each category has an equal number of examples (150 training and 150 test samples). The ratio between training and testing samples employed in our experiment was the same as that in [57].

#### 4.1.3 Evaluation method

To evaluate the classification performance of the deep learning models objectively, the indicators of [33] were adopted, namely precision, recall, accuracy, F1-score, AP, and mAP. Precision is the ratio between the true positive samples and the total number of samples predicted as positive, while recall is the ratio of true positive samples predicted compared to all the positive samples. The F1-score is the harmonic mean of precision and recall. Accuracy refers to the ratio of the number of correctly predicted samples to the total number of samples. AP refers to the average value of the recall rate and ranges from 0 to 1. The mAP is the arithmetic average of all AP. The specific equations of these metrics are shown in Table 2. In Table 2, TP, FP, TN, and FN refer to the number of true positives, false positives, true negatives, and false negatives, respectively.

**Table 2:** Evaluation metrics for EM image classification.

Assessments	Formula
Precision (*P*)	$\frac{TP}{TP+FP}$
Recall (*R*)	$\frac{TP}{TP+FN}$
F1-score	$2\times \frac{P\times R}{P+R}$
Accuracy	$\frac{TP+TN}{TP+TN+FP+FN}$
AP	$\frac{1}{M}\sum_{i=1}^{M}{\text{Precision}}_{\max}(i)$
mAP	$\frac{1}{K}\sum_{j=1}^{K}AP(j)$

#### 4.1.4 Implementation details

The experiments were performed on an NVIDIA Quadro RTX 4000 GPU and the same training strategy used in [33] was adopted. Specifically, the learning rate was set to 0.002 and the batch size was set to 32. The model was trained for 100 epochs. All images were cropped to 224×224 pixels.

### 4.2 Experimental results and analysis

#### 4.2.1 Classification performance on EMDS-6

**Fig 4:**
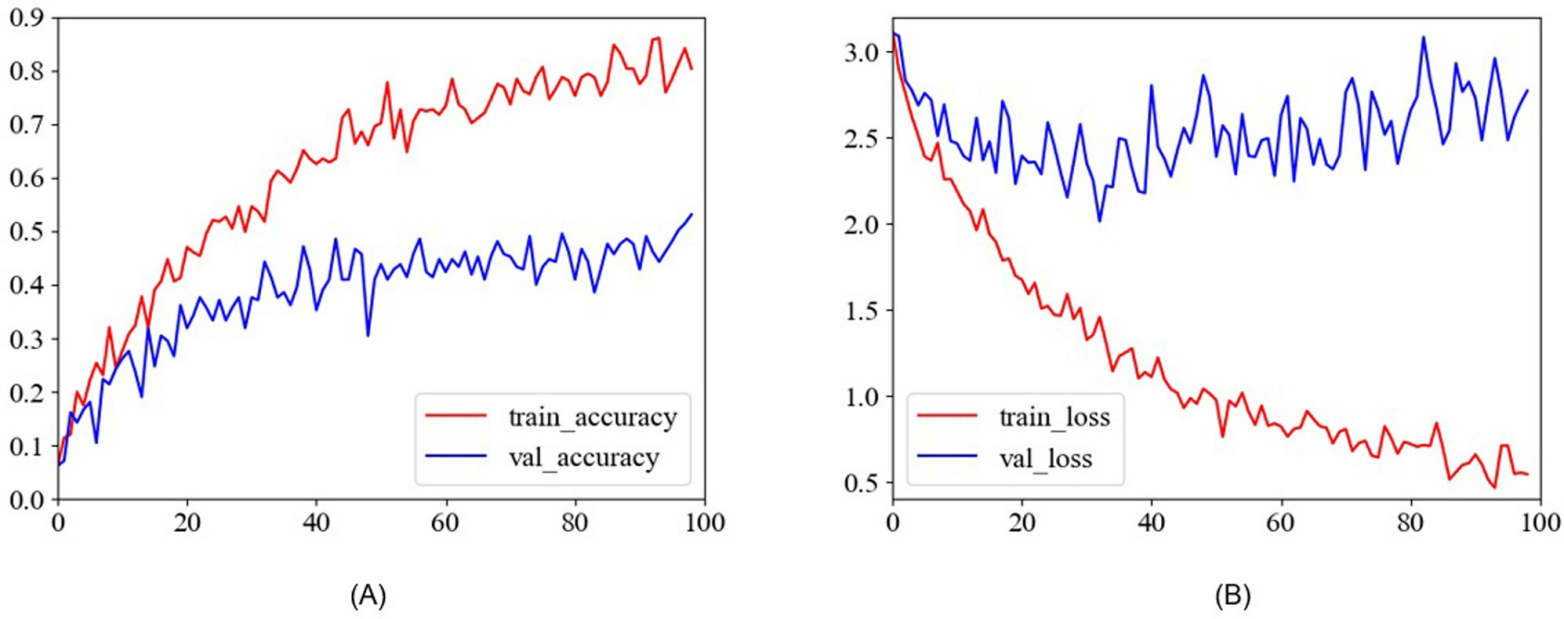
The accuracy and loss curves of the proposed HTEM model.

The accuracy and loss curves of HTEM on EMDS-6 are shown in Fig 4. In Tables 3 and 4, we also evaluate the effectiveness of the proposed HTEM models in comparison to state-of-the-art classification models, including transformer-based models (ViT, DeiT, T2T-ViT, and BotNet) and representative CNN-based models (Xception, ResNet18, ResNet34, MobileNetV2, Googlenet, Densenet121, Densenet169, and VGG11). All the other models’ implementations were obtained from [33].

**Table 3:** Comparison of classification results of different models on the validation set.

Model	*R*(%)	*P*(%)	F1_score(%)	Accuracy(%)	Params Size (MB)	Time(s)
BotNet	30.48%	32.61%	30.06%	30.48%	72.2	894
VGG11	31.43%	41.20%	29.97%	31.43%	491	864
T2T-ViT	34.29%	38.17%	34.54%	34.28%	15.5	825.3
ViT	37.14%	41.02%	35.95%	37.14%	31.2	715
Deit	39.05%	39.37%	37.70%	39.05%	21.1	817.27
MobileNetV2	39.52%	39.57%	37.01%	39.52%	8.82	767
ResNet18	40.95%	45.55%	41.05%	40.95%	42.7	739
Densenet169	40.95%	43.62%	39.89%	40.95%	48.7	988
Densenet121	40.95%	43.61%	40.09%	40.95%	27.1	922
Googlenet	41.90%	42.83%	40.49%	41.91%	21.6	772
ResNet34	42.86%	45.33%	42.31%	42.86%	81.3	780
Xception	45.71%	52.48%	44.95%	45.71%	79.8	996
HTEM	**53.00**%	**56.82**%	**53.33**%	**53.00**%	**1.3**	**342**

P denotes Precision, and R denotes Recall.

**Table 4:** Comparison of classification results of different models on the test set.

Model	*R*(%)	*P*(%)	F1_score(%)	Accuracy(%)	Params Size (MB)	Time(s)
BotNet	25.40%	29.65%	26.04%	25.39%	72.2	6.5
VGG11	27.61%	29.64%	26.00%	27.62%	491	4.98
ViT	31.75%	33.84%	31.47%	31.74%	31.2	3.77
Deit	32.39%	34.40%	32.74%	32.38%	21.1	5.43
Densenet121	33.01%	39.20%	33.79%	33.02%	27.1	9.27
ResNet18	33.33%	38.10%	32.36%	33.33%	42.7	4.92
Densenet169	33.65%	36.55%	33.79%	33.65%	48.7	11.13
T2T-ViT	34.29%	38.17%	34.54%	34.28%	15.5	4.44
MobileNetV2	34.29%	38.21%	33.07%	34.29%	8.82	5.13
Googlenet	35.23%	37.70%	34.21%	35.24%	21.6	5.97
ResNet34	36.51%	42.92%	36.22%	36.51%	81.3	6.14
Xception	40.33%	49.71%	41.41%	40.32%	79.8	5.63
HTEM	**47.02**%	**48.81**%	**46.72**%	**47.02**%	**1.3**	**3.12**

P denotes Precision, and R denotes Recall.

As shown in Fig 4A, the accuracy on the training set is significantly higher than that on the validation set. The accuracy of the HTEM model training set increases rapidly, approaching its highest point of 90% after 80 epochs. After 40 epochs, the accuracy on the validation set approaches 50%, which is its maximum. As shown in Fig 4B, after 80 epochs, the HTEM training set loss curve begins to gradually taper out toward its minimum value. However, in the validation set, this occurs after only 30 epochs.

As shown in Table 3, we compared the proposed HTEM model with the other models on the validation set of EMDS-6. Compared to the CNN-based models, HTEM achieves an accuracy of 53.00%, which is higher than Xception, ResNet34, and GoogleNet by 7.29, 10.14, and 11.09 percentage points (pp.), respectively. Compared with Densenet121, Densenet169, ResNet18, MobileNetV2, and VGG11, HTEM even exceeds 10pp. in classification accuracy improvement. Similarly, HTEM achieved the highest recall, precision, and F1-score values in the validation set results, which are 53.00%, 56.82%, and 53.33%, respectively, and outperformed all the other models on EMDS-6. This is a surprising result since the size of HTEM is one-sixtieth the size of the Xception model. Compared to the transformer-based models, our model achieves greater accuracy over DeiT, ViT, T2T-ViT, and BotNet by 13.95, 15.86, 18.72, and 22.52pp., respectively, while the number of parameters is only one-twelfth that of T2T-ViT.

To further illustrate the performance of the HTEM model proposed in this paper, we again compared all models on the test set of EMDS-6, as shown in Table 4. On the test set, the performance of all models decreased compared to that on the validation set because the test set comprised image data that were unknown to the models, and because of the performance of the test set reflects the models’ generalization ability. Compared to the models on the test set, the proposed HTEM model still achieved the highest recall, precision, F1-score, and accuracy results, at 47.02%, 48.81%, 46.72, and 47.02%, respectively. It should be noted the HTEM model required the least training time and performed inference faster than the other models, while still achieving higher accuracy on EMDS-6, as shown in Tables 3 and 4. The experimental results show that HTEM’s transformer configuration can be trained to achieve high accuracy on EMDS-6 through the proposed hierarchical structure and incorporation of local feature information. This outcome is consistent with this study’s main objective.

#### 4.2.2 Classification performance on WHOI

To further validate the effectiveness of our proposed model on a different EM dataset, we evaluated the proposed HTEM on the publicly available dataset used in [57], which reports classification benchmarks on the WHOI plankton datasets. The accuracy and F1_score of our model were compared with that of the best models in [57]. As shown in Table 5, the HTEM model outperformed all the previous methods. the proposed HTEM demonstrated an accuracy of 2.5pp. and 1.7pp. compared to individual CNN models, i.e., InceptionV3 and EfficientNet B7. Compared to the ensemble models, i.e., Best_6_avg and Best_6_stack, HTEM showed slight improvements in terms of accuracy and F1-score. The experimental results show that the proposed HTEM can achieve the desired classification performance on the WHOI dataset, further validating the rationality of our model design.

**Table 5:** Comparison of classification results of different models on the WHOI dataset.

Model	Accuracy(%)	F1_score(%)
InceptionV3	96.5%	89.3%
EfficientNet	97.3%	91.6%
Best_6_avg	97.6%	93.0%
Best_6_stack	97.7%	92.5%
HTEM	**99.0**%	**94.2**%

### 4.3 Extended experiment

#### 4.3.1 Classification performance on EMDS-6 after data augmentation

We compared the proposed HTEM model with all the models on the EMDS-6 after data augmentation, as shown in Tables 6 and 7. All the other models’ implementations were obtained from [33]. On the validation set, although all the CNN-based models’ performance was improved after data augmentation, the proposed HTEM model still outperformed the second-best Xception on its recall, precision, F1-score, and accuracy values by 6.93, 9.48, 8.01, and 6.93pp., respectively. In addition, it also outperformed the Xception network in terms of training time, as it required only one-third of Xception’s time. Compared with ResNet18, the proposed HTEM model has a similar training time but its accuracy is superior by 15.11pp. Compared with MobileNetV2, VGG11, ResNet34, Googlenet, Densenet121, and Densenet169, HTEM achieves the best classification performance with the least training time in all cases.

**Table 6:** Comparison of classification results of different models on the validation set after data augmentation.

Model	*R*(%)	*P*(%)	F1_score(%)	Accuracy(%)	Params Size (MB)	Time(s)
T2T-ViT	35.56%	38.43%	36.19%	35.56%	15.50	1385.62
BotNet	36.59%	36.38%	35.59%	36.59%	72.2	2000.17
ViT	39.05%	43.50%	38.52%	39.05%	31.20	902.27
Densenet169	42.14%	48.04%	42.79%	42.14%	48.70	2526.61
Densenet121	42.38%	46.91%	42.39%	42.38%	27.10	2169.11
Deit	43.34%	46.62%	43.29%	43.33%	21.10	1306.99
Googlenet	44.29%	47.16%	43.50%	44.29%	21.60	1257.33
ResNet18	44.44%	51.87%	43.03%	44.44%	42.70	1090.39
ResNet34	46.10%	47.85%	44.68%	46.11%	81.30	1335.87
VGG11	48.10%	52.40%	48.44%	48.10%	491.00	1745.73
MobileNetV2	49.67%	51.91%	48.82%	49.68%	8.82	1237.49
Xception	52.62%	52.05%	50.63%	52.62%	79.80	2636.08
HTEM	**59.55**%	**61.53**%	**58.64**%	**59.55**%	**1.18**	**901.31**

P denotes Precision, and R denotes Recall.

**Table 7:** Comparison of classification results of different models on the test set after data augmentation.

Model	*R*(%)	*P*(%)	F1_score(%)	Accuracy(%)	Params Size (MB)	Time(s)
ViT	28.58%	29.63%	27.86%	28.57%	31.2	3.72
T2T-ViT	30.48%	35.88%	30.85%	30.48%	15.50	5.41
Deit	32.39%	34.40%	32.74%	32.38%	21.1	4.41
BotNet	36.50%	39.12%	36.35%	36.51%	72.2	6.44
VGG11	37.14%	38.81%	36.70%	37.14%	491	4.96
Densenet169	37.14%	41.51%	37.37%	37.14%	48.7	11.04
Googlenet	37.46%	43.55%	37.92%	37.46%	21.6	6.03
ResNet34	38.73%	42.25%	37.84%	38.73%	81.3	6.07
Densenet121	38.73%	40.28%	38.20%	38.73%	27.1	8.98
ResNet18	39.05%	44.82%	39.22%	39.05%	42.7	4.90
MobileNetV2	42.54%	47.56%	43.07%	42.54%	8.22	5.04
Xception	45.71%	50.43%	46.15%	45.71%	79.8	5.49
HTEM	**54.73**%	**57.52**%	**54.91**%	**54.73**%	**1.18**	**3.02**

P denotes Precision, and R denotes Recall.

Compared to the transformer-based models, the performance of T2T-ViT, ViT, and Deit did not improve significantly after data augmentation. This is because the geometric augmentation has a greater impact on local dependencies and does not affect long-range dependencies. The BotNet model had a greater performance improvement because it introduces the attention module of the transformer in the ResNet network, which has the ability to capture both long-range dependencies and local information. However, BotNet still fell behind the HTEM in terms of accuracy by 22.96pp. This is because the proposed HTEM model introduces more convolution operations in the transformer and can capture both long-range dependencies and a large amount of local features at the same time, so data augmentation results in substantial performance gains.

On the test set, HTEM achieved an accuracy of 54.73%, which is higher than Xception, MobileNetV2, and ResNet18 by 9.02pp., 12.19pp., and 15.68pp., respectively. Similarly, it achieved 54.73%, 57.52%, and 54.91% in its recall, precision, F1-score and outperformed the aforementioned transformer-based and CNN-based models. As shown in Table 6, the accuracy of the Xception model decreased by 6.91%, while the HTEM model decreased by only 4.82%. This is due to the fact that HTEM inherits the strong generalization ability of the transformer. In addition, although the amount of data increased, the HTEM model still had the smallest training and inference times. The experimental results show that HTEM benefits from the advantages of both CNNs and transformers at the same time, further validating the rationality and effectiveness of the proposed model’s design.

#### 4.3.2 Statistics analysis on EMDS-6

For the experiments presented in this section, the EMDS-6 dataset was randomly divided three times to obtain training, validation and test sets. The effectiveness of the proposed HTEM models was also evaluated in comparison to state-of-the-art classification models, including transformer-based models (ViT and BotNet) and representative CNN-based models (Xception, ResNet18, ResNet34, and MobileNetV2). All the other models’ implementations were obtained from [33]. The average results of the three experiments are shown in Table 8, where it can be seen that under the original dataset, the proposed HTEM model has the highest classification performance of all the models. After data augmentation, HTEM still yields the highest classification performance, outperforming Xception, MobileNetV2, ResNet34, ViT, and BotNet by 8.95pp., 8.96pp., 14.03pp., 23.02pp., and 20.69pp., respectively. The classification performance of all models was improved except for ViT. This is because the pure transformer model is insensitive to geometric transformation-based data augmentation. These experimental results further verify the superiority and effectiveness of the model proposed in this paper.

**Table 8:** Comparison of different deep learning models on EMDS-6 test sets. (In [%]).

Model	Original Data				Augmented Data			
	*R*(%)	*P*(%)	F1_score(%)	Accuracy(%)	*R*(%)	*P*(%)	F1_score(%)	Accuracy(%)
BotNet	29.00	31.11	28.46	28.99	33.02	34.29	32.45	33.02
ViT	33.24	34.92	32.63	33.23	30.69	32.49	30.08	30.69
ResNet34	37.14	41.96	36.93	37.14	39.68	43.15	39.54	39.68
MobileNetV2	34.50	37.24	33.86	34.50	44.75	48.31	44.82	44.75
Xception	39.37	44.25	39.07	39.37	44.76	47.97	44.53	44.76
HTEM	**47.01**	**48.56**	**46.05**	**47.01**	**53.71**	**57.31**	**53.45**	**53.71**

P denotes Precision, and R denotes Recall.

#### 4.3.3 Feature information analysis

**Fig 5:**
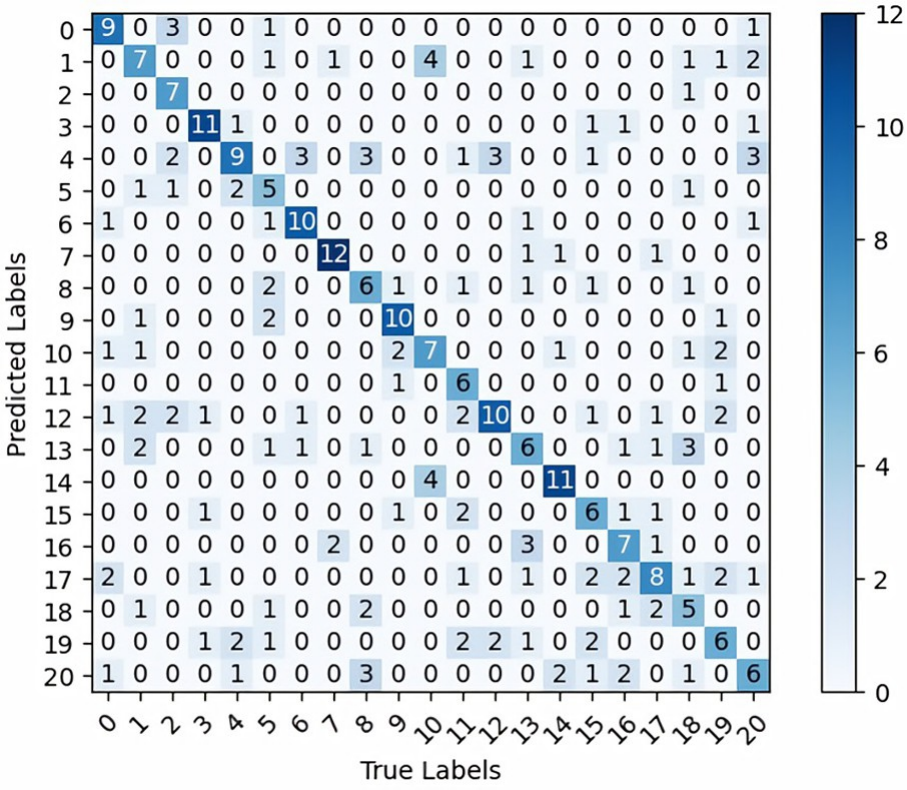
Confusion matrix of HTEM model on the test set after data augmentation. In the confusion matrix, 0, 1, 2, 3, 4, 5, 6, 7, 8, 9, 10, 11, 12, 13, 14, 15, 16, 17, 18, 19, 20 represent Actinophrys, Arcella, Aspidisca, Codosiga, Colpoda, Epistylis, Euglypha, Paramecium, Rotifera, Vorticella, Noctiluca, Ceratium, Stentor, Siprostomum, K. Quadrala, Euglena, Gymnodinium, Gymlyano, Phacus, Stylongchia, Synchaeta.

**Fig 6:**
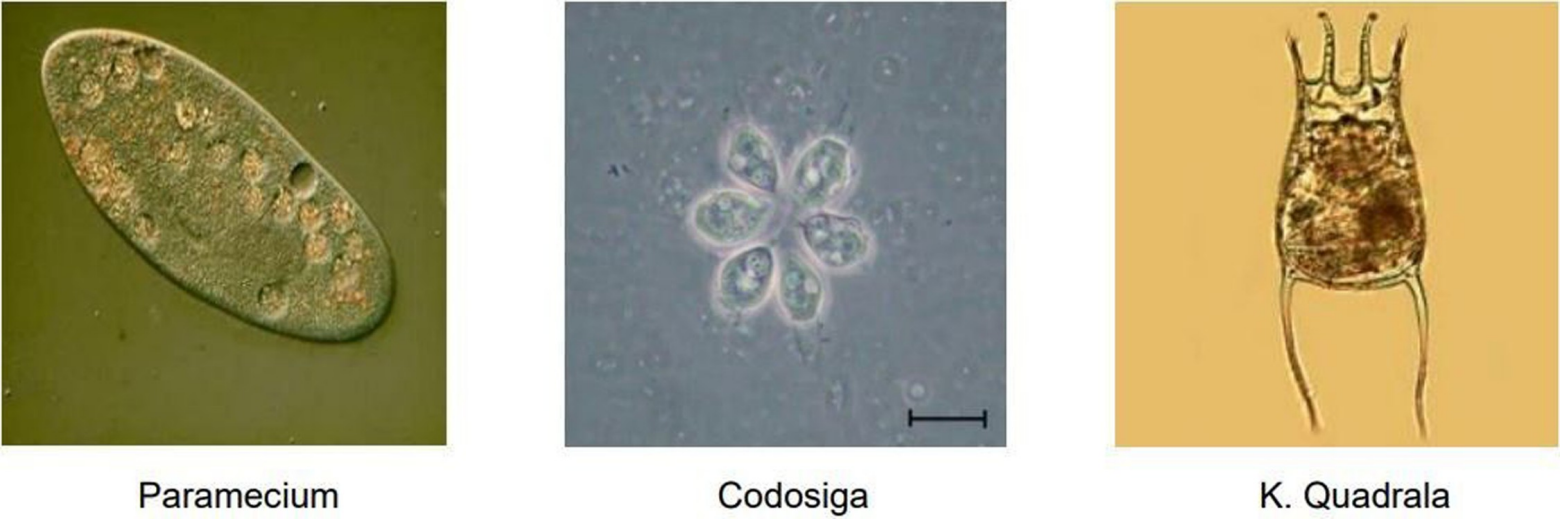
Images of Paramecium, Codosiga, and K. Quadrala.

**Fig 7:**
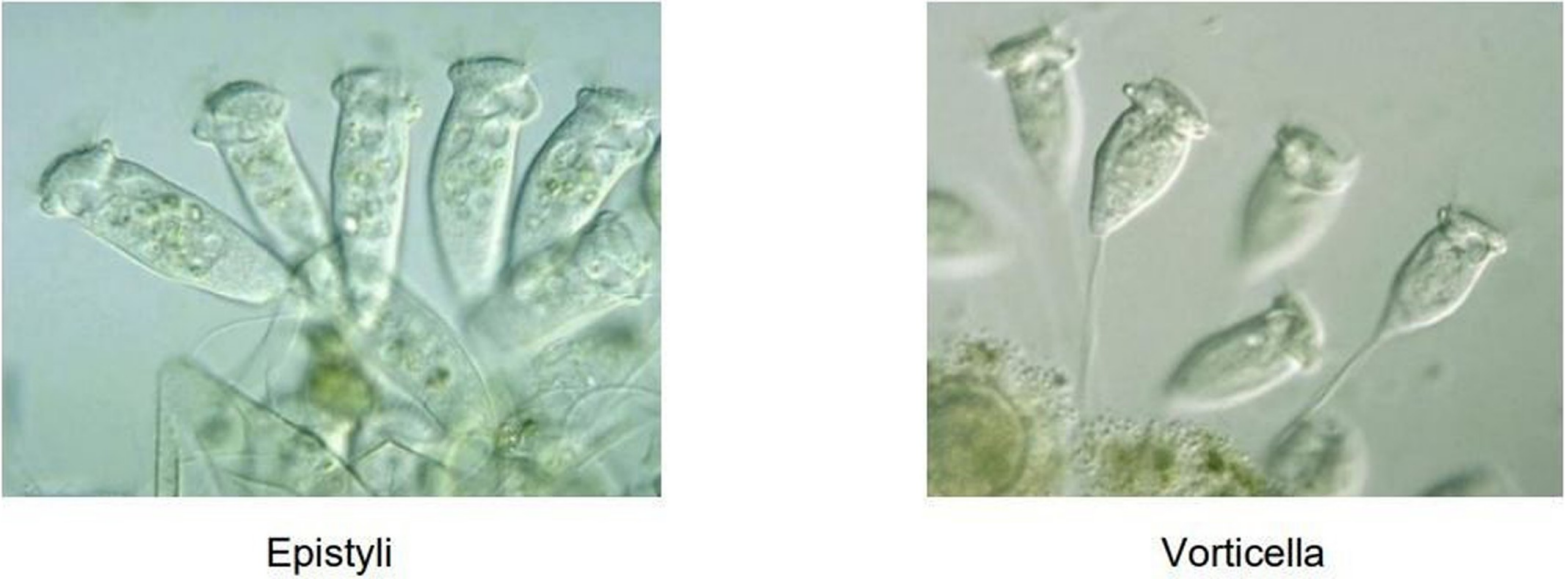
Images of Epistylis, and Vorticella.

Fig 5 presents the confusion matrix of the HTEM model regarding the EM images on the test set of EMDS-6 after data augmentation. 164 out of the total of 315 EM images were classified into the correct category. The HTEM model performed well on the Paramecium, Codosiga, and K. Quadrala image classification because these three EMs have more obvious and less confusing characteristics, as shown in Fig 6. The predictions of the Epistylis and Phacus classes were slightly inaccurate, as evidenced by the result that 10 out of the 15 Epistylis images were identified incorrectly. The misclassification was usually caused by the very similar features of the two EMs, as shown in Fig 7, causing the Epistylis images to be easily confused for Vorticella images. In fact, these EM images are very difficult for non-experts to distinguish. In summary, the proposed HTEM model can assist in the task of EM classification, but for EMs with particularly similar features, further confirmation of the categories by professionals using appropriate instrumentation is still required.

#### 4.3.4 Imbalanced training on EMDS-6

In this section, to validate the classification ability of the proposed HTEM model on imbalanced datasets, the EMDS-6 dataset is restructured. The procedure was repeated 21 times and 21 different unbalanced EMs datasets were obtained. The specific data reorganization method is shown in Section 4.1.2. The AP of the deep learning models was calculated after training on each dataset version. Table 9 shows the AP and mAP for each model on the validation set. CNN- and transformer-based models such as ResNet50, Inception-V3, Xception, and ViT were selected respectively for comparison. All the other models’ implementations were obtained from [33]. It is evident from Table 9 that the mAP of the HTEM model is the highest, at 60.50%. HTEM obtains the highest AP on the 10th dataset version, with an AP of 81.56%, and the lowest AP on the 16th dataset, at 41.24%. Compared to the CNN-based models, the proposed model achieves greater accuracy over Xception, Inception-V3, and ResNet50 by 3.89, 17, and 19.47pp., respectively. This is mainly due to HTEM’s ability to capture both local information and long-range dependencies. In addition, it should be noted that HTEM’s performance constitutes a significant improvement over the ViT model, outperforming by 25.57pp. This is because of the greatly improved ability of the model to acquire local features via the convolution operations integrated in the transformer model. The experimental results show that HTEM can achieve high classification performance on the imbalanced versions of the EMDS-6 dataset, further validating its effectiveness.

**Table 9:** AP and mAP of different models in imbalanced training. (In [%]).

Model	1	2	3	4	5	6	7	8	9	10	11
ViT	30.77%	44.99%	18.43%	48.51%	74.47%	76.17%	50.98%	15.32%	31.12%	60.74%	54.02%
ResNe50	30.58%	45.96%	14.24%	68.19%	66.15%	43.10%	71.24%	46.51%	31.87%	62.19%	36.79%
Inception-V3	37.75%	36.79%	33.41%	56.37%	55.77%	43.51%	59.52%	41.18%	38.40%	75.03%	69.26%
Xception	37.66%	51.16%	29.72%	68.32%	73.66%	67.96%	79.19%	65.41%	55.84%	82.97%	55.91%
HTEM	56.04%	51.23%	46.95%	71.54%	72.21%	65.56%	80.02%	68.48%	49.35%	81.56%	56.59%
Model	12	13	14	15	16	17	18	19	20	21	mAP
ViT	15.24%	17.84%	25.46%	6.74%	13.95%	48.61%	7.26%	60.33%	23.07%	9.53%	34.93%
ResNe50	15.59%	42.12%	68.57%	24.94%	17.49%	47.52%	6.64%	49.04%	16.73%	56.10%	41.03%
Inception-V3	15.09%	49.09%	64.11%	37.91%	15.00%	43.98%	15.84%	54.40%	10.78%	60.38%	43.50%
Xception	54.16%	52.28%	65.06%	46.36%	30.61%	60.41%	31.21%	61.14%	45.50%	74.36%	56.61%
HTEM	53.22%	53.02%	71.54%	70.00%	41.24%	58.06%	38.66%	62.35%	49.75%	73.21%	**60.50**%

#### 4.3.5 Classification performance of EMDS-6 test set in the presence of noise

In this section, the classification performance of the proposed HTEM model in the presence of noise is verified. To achieve this, salt-and-pepper and Gaussian noise was added to the images of the EMDS-6 test set and the latter was input to the model trained on the clear images to verify the model’s generalization ability in the presence of noise. We report the results in Table 10. We also evaluated the accuracy of the proposed HTEM models in comparison to state-of-the-art classification models, including transformer-based models (ViT, DeiT, and T2T-ViT) and representative CNN-based models (Xception, ResNet18, ResNet34, MobileNetV2, and Googlene). To better illustrate the effect of noise on the classification accuracy of the models, we used the classification accuracy of each model on the test set in the absence of noise as a benchmark. As shown in Table 10, the proposed HTEM model again achieved the highest classification accuracy on the noisy test set, achieving 46.67% and 46.07% in the salt-and-pepper and Gaussian noise cases, respectively. In addition, it is evident that noise has a greater impact on the CNN-based models than on the transformer-based models. This is because transformer-based models have better generalization ability than CNN-based models, which mainly rely on local features. The proposed HTEM, due to the combined advantages of both CNNs and transformers, retains its high feature extraction capability in the presence of noise interference, further validating the rationality and effectiveness of the proposed model.

**Table 10:** Comparison of classification accuracy of different models on the test set in the presence of noise.

Model	No noise	Pepper-salt noise	Gaussian noise
ViT	31.74%	30.83% (-0.91)	30.03% (-1.71)
Deit	32.38%	31.65% (-0.73)	30.75% (-1.63)
ResNet18	33.33%	27.31% (-3.12)	28.69% (-4.64)
T2T-ViT	34.28%	30.21% (-0.72)	32.39% (-1.89)
MobileNetV2	34.29%	31.27% (-3.02)	30.35% (-3.94)
Googlenet	35.24%	32.63% (-2.61)	31.25% (-3.99)
ResNet34	36.51%	33.95% (-2.56)	32.78% (-3.73)
Xception	40.32%	37.66% (-2.66)	36.80% (-3.52)
HTEM	**47.02**%	**46.67% (-0.35)**	**46.07% (-0.95)**

P denotes Precision, and R denotes Recall.

### 4.4 Ablation study

To confirm the viability of the proposed blocks introduced as novel transformer architectural elements, five ablation studies were performed. First, the effect of the CPSA and LFFN blocks’ presence on the HTEM’s performance on EMDS-6 was analyzed. Then, it is demonstrated that the position embeddings can be dropped from the model. Finally, the impact of the configuration of three key parameters on network performance is discussed.

#### 4.4.1 Effectiveness of CPSA and LFFN on EMDS-6 test set after data augmentation

The proposed HTEM model differs in a number of ways from transformer-based models like ViT, the most notable difference being the proposed CPSA and LFFN blocks. In the first experiment, every block in the model was replaced by a transformer block while maintaining the other parameters the same to investigate the effects of CPSA and LFFN on the performance of the proposed HTEM. The modified model’s classification accuracy was then obtained on the augmented EMDS-6 dataset. The model was trained for 100 epochs. The effects of each modification on the proposed HTEM model are presented in Table 11. It is evident that the CPSA block has a greater impact on the classification accuracy of small EM datasets, while the LFFN block also brings about a significant improvement on the classification effect compared to the FFN of the original transformer. These findings suggest that adding CPSA and LFFN to the transformer can help improve the classification accuracy in the case of small datasets like EMDS-6.

**Table 11:** Ablations on CPSA and LFFN.

Model	None	CPSA	LFFN	Accuracy(%)
HTEM	√			34.61%
		√		47.82%
			√	45.35%
		√	√	**54.73%**

#### 4.4.2 Removal of position embedding on EMDS-6 test set after data augmentation

Given the increased local information integrated into the HTEM model, the necessity of the position embedding was analyzed. The results in Table 12 show that omitting the position embedding has no negative effects on the proposed model’s performance. Therefore, the final proposed configuration of the model omits the position embeddings. It should be noted that since the T2T-ViT cannot capture more local features, a corresponding change of removing the position embedding results in a performance loss of 4.12% on the EMDS-6. This further demonstrates the effectiveness of the proposed HTEM. Since position embedding needs to be implemented using a fixed-length learnable vector according to the size of the input image, this greatly limits the model’s ability to adapt to inputs of different sizes. CPVT [58] tries to use conditional position encoding to represent position embeddings to help transformer during training. The HTEM can forego the position embedding completely, which constitutes a new approach.

**Table 12:** Ablations on position embedding.

Model	PE	Accuracy(%)
T2T-ViT	√	**30.48%**
	×	26.36%
HTEM	√	54.14%
	×	**54.73%**

#### 4.4.3 Different types of layout of each stage on EMDS-6 validation set

As CPSA and LFFN are the two core blocks of the HTEM model, an interesting question is how to determine the optimal number of these two blocks to achieve the best classification performance. In each stage of the HTEM model, we used the same number of CPSA and LFFN blocks to form a block unit, so this question is equivalent to determining the number of modular units in each stage, which is called a "layout" design. For this purpose, several different layouts were compared and the results are presented in Table 13. By selecting several common layout designs in each stage, it was found that the model with the [2, 2, 4, 2] layout achieved the optimal classification accuracy. When the number of block units was too small, the salient features of the images could not be learned, while high classification accuracy also cannot be obtained with too many block units due to the small size of the EMDS-6 dataset.

**Table 13:** Ablations study results on different types of layout of each stage.

Model	Layout	Accuracy(%)
HTEM	[2,2,2,2]	44.34%
	[3,3,6,3]	41.26%
	[4,4,8,4]	37.56%
	[2,2,4,2]	53.00%

#### 4.4.4 Different types of CPSA block on EMDS-6 validation set

In the CPSA module, each attention head learns image features of different dimensions, while the convolution operations improve image local feature acquisition. A reasonable configuration of attention heads and convolutional reduction rates can potentially improve the accuracy of the model. Therefore, appropriate experiments were performed to study the effect of different head and reduction rate configurations. It was found that using progressively changing heads and reduction rates at each stage improves the model performance effectively, as shown in Table 14.

**Table 14:** Ablations study results on different configurations of attention heads and reduction rates.

Model	Heads Numbers	reduction rates	Accuracy(%)
HTEM	[3,3,3,3]	[2,2,2,2]	42.55%
	[8,8,8,8]	[2,2,2,2]	39.25%
	[3,3,3,3]	[8,4,2,1]	46.56%
	[1,2,4,8]	[8,4,2,1]	53.00%

#### 4.4.5 Different types of LFFN block on EMDS-6 validation set

In the LFFN block, the size of the kernel determines the size of the captured local information. Therefore, kernel sizes of 1×1, 3×3, and 5×5 were tested and the results are presented in Table 15. The 1×1 type shows poor performance compared to the baselines that do not employ depth-wise convolution as an extension layer. This demonstrates that increasing the number of layers in the transformer will not necessarily result in improvement. When the kernel size is increased, the block captures more local content, and corresponding gains were improved for both the 3×3 and 5×5 sizes. However, the kernel size of 3×3 was selected as the default option, as it achieved the best trade-off between the training time and model accuracy.

**Table 15:** Ablations study results on the type of LFFN block.

Model	Kernel Size	Accuracy(%)	Time(s)
HTEM	X	49.59%	290
	1×1	47.56% (-2.03)	315
	3×3	53.00% (+3.41)	342
	5×5	53.04% (+3.45)	417

## 5. Conclusion

In this work, we have presented a hybrid architecture comprising a transformer and a CNN model, which is suitable for EM image classification tasks on small datasets. A series of studies were conducted to investigate various components and the hierarchical architecture design and the introduction of the CPSA and LFFN blocks was demonstrated as a critical factor for the improved performance of the HTEM model. The model exhibits better performance than previous works on the EMDS-6 and WHOI with accuracies of 47.02% and 99%, respectively, while maintaining computational efficiency. Furthermore, the proposed model no longer requires position embedding, as it can capture more local features due to the introduction of the two blocks. By incorporating rational designs, we provide a new perspective for EM image classification.

Although our model achieves high classification performance on small EM datasets, its applicability to other types of image datasets still needs further study. In future work, the proposed model will be applied to more image datasets such as facial image datasets to further validate its generalization performance.
